# Associations of Mentally Active and Passive Sedentary Behavior with Sleep Quality and Duration in Pregnant Women of Advanced Versus Younger Maternal Age

**DOI:** 10.3390/jcm14248666

**Published:** 2025-12-07

**Authors:** Abdullah Bandar Alansare

**Affiliations:** Department of Exercise Physiology, College of Sport Sciences and Physical Activity, King Saud University, King Khalid Rd, B69-G1 Building, Riyadh 80200, Saudi Arabia; aalansare@ksu.edu.sa; Tel.: +966-555-061381

**Keywords:** high-risk pregnancy, midwifery, prenatal health, prolonged sitting, weekends, weekdays

## Abstract

**Background/Objectives**: To examine associations of mentally active and passive sedentary behavior (SB) with sleep quality and duration in pregnant women of advanced (AMA) and younger (YMA) maternal age, separately, and evaluate effects of SB patterns (weekends vs. weekdays). **Methods**: This secondary analysis of an observational, clinic-based, cross-sectional study included pregnant women of AMA (*n* = 225; 37.8 ± 2.6 years) and YMA (*n* = 710; 27.5 ± 3.8 years) from any trimester. SB and sleep were assessed using the Arabic version of the Sedentary Behavior Questionnaire and the Pittsburgh Sleep Quality Index, respectively. Logistic regression models evaluated associations of maternal mental activity-based SB with sleep outcomes. **Results**: Higher mentally passive SB on weekdays was unfavorably associated (odds ratios ranged between 1.58 and 2.12; *p* < 0.05 for all), and on weekends was paradoxically and favorably associated (odds ratios ranged between 0.53 and 0.62; *p* < 0.05 for all) with sleep quality only in pregnant women of AMA. Higher mentally passive SB across the week or on weekdays was unfavorably associated (odds ratios ranged between 1.11 and 1.65; *p* < 0.05 for all), while higher mentally passive SB on weekends and mentally active SB across the week or on weekends were paradoxically and favorably associated (odds ratios ranged between 0.57 and 0.91; *p* < 0.05 for all) with a higher adherence to sleep duration recommendations in both pregnancy groups. **Conclusions**: These findings suggest that some relationships between mental activity-based SB and prenatal sleep health may vary across maternal age groups. The cross-sectional design limits causal inference, emphasizing the need for longitudinal and randomized studies on mental activity-based SB and sleep health in pregnant women of AMA and YMA.

## 1. Introduction

Globally, poor sleep quality is not only prevalent during pregnancy (44.5%) but also carries over into the postpartum period (67.2%) [1]. A large systematic review and meta-analysis, which included more than 58 million pregnant women worldwide, revealed that impaired sleep health during pregnancy was associated with greater odds of adverse maternal and fetal complications, including pre-eclampsia, gestational hypertension and diabetes, cesarean section, preterm birth, larger for gestational age, and stillbirth (odds ratios ranged between 1.25 and 2.80) [2]. As such, poor parental sleep health is recognized as a significant public health challenge, prompting a call to examine evidence-based strategies [3,4]. Apart from the risks posed by sleep medications on maternal and fetal outcomes [5], pregnant women and their partners particularly indicated a preference for non-pharmacological to pharmacological interventions to enhance pregnancy-related sleep disorders [6].

Despite being largely overlooked, the relationships between poor maternal sleep health and pregnancy complications appeared to be more evident among pregnant women of advanced maternal age (AMA) (i.e., ≥35 years old) compared to those of younger maternal age (YMA) (i.e., <30 years old) [7,8]. These variations may be partially explained by pregnant women of AMA having lower melatonin levels [9] and elevated inflammation [8], both can contribute to greater sleep disruption. Alarmingly, there is a worldwide troubling upward trend in maternal age at first pregnancy [10,11]. Together with the differing impacts of poor sleep health on pregnancy complications among those of AMA vs. YMA, the need to investigate non-pharmacological strategies to optimize prenatal sleep health, designed for each age group separately, becomes increasingly imperative.

Sedentary behavior (SB) (i.e., any waking behavior in seated, lying, or reclining posture with minimal energy expenditure) [12] is highly prevalent among pregnant women [13]. Aside from its broad relationships with pregnancy complications across all pregnancies [13], higher SB was recently linked to a higher risk of premature rupture of the membrane in pregnant women of AMA [14]. Notably, there is a paucity of research examining the associations of SB with sleep health during pregnancy, with only two published studies identified. An SB reduction intervention trial in pregnant women (18 to 45 years old) successfully reduced total SB by 0.84 h/day, yet interestingly decreased sleep quality (i.e., worse sleep efficiency) [15]. The other was a cross-sectional study which observed complex paradoxical associations of total, domains (i.e., leisure, occupational, commuting), and patterns (i.e., weekdays vs. weekends) of SB with sleep quality and duration in pregnant women (≥18 years old) [16]. These earlier investigations have highlighted that maternal age and SB influence prenatal sleep health in complex ways. As such, these studies have called for further research to examine the associations of SB with prenatal sleep health beyond total duration or weekly averages. Such research should also analyze pregnant women of AMA and YMA separately, to better inform lifestyle modification strategies aimed at optimizing maternal sleep health.

Of particular relevance, an integrated framework for SB assessments was recently proposed to gain deeper insights into how different SB affect health [17]. Beyond the typical SB domain classifications, this framework advocates classifying SB by its mental activity, defining it as mentally active when it requires high cognitive effort and mentally passive when it requires low cognitive effort [17]. This distinction is crucial as growing evidence shows that the context of SB drives its health risks [17,18,19]. The influence of mental activity-based SB on sleep health has been previously investigated in diverse populations [18,19,20,21], but not in pregnant women. In contemporary lifestyles, mentally active and passive SB (e.g., TV viewing, playing video games, social media scrolling, or reading) are common among individuals, including pregnant women. Given its modifiability, mental activity-based SB represents a promising lifestyle target for non-pharmacological interventions to improve maternal sleep health. Therefore, this secondary analysis examined the associations of mentally active and passive SB with sleep quality and duration in pregnant women of AMA and YMA, separately. The study also evaluated how patterns (i.e., weekend vs. weekday) of SB influence these relationships.

## 2. Materials and Methods

This study adhered to the Strengthening the Reporting of Observational Studies in Epidemiology (STROBE) guidelines. Approval of the study protocol was obtained from the Institutional Review Board at King Saud University (No.: KSU-HE-23-516; 21 May 2023).

### 2.1. Study Design, Recruitment, and Participant Selection

This investigation was a secondary analysis of an observational, clinic-based, cross-sectional study conducted at a single point in time for each pregnant woman between 3 July 2023, and 24 August 2023. The study protocol was detailed elsewhere [22]. Briefly, using a convenience recruitment strategy, trained research team members visited public and private obstetrics and gynecology clinics in six major cities in Saudi Arabia and interviewed potential participants. Eligibility for the study enrollment required that women be currently pregnant and permanent residents of Saudi Arabia. Eligible women provided informed consent and were subsequently interviewed by a trained research team member using questionnaires, collecting self-reported data. Initially, 952 pregnant women participated in the study. Of them, 17 participants were omitted from the current analysis due to unreasonable reports (≥24 h/day) of physical activity (PA) (*n* = 11), SB (*n* = 2), sleep (*n* = 2), weeks of pregnancy (e.g., 63 weeks) (*n* = 1), or missing weight and/or height data (*n* = 1). Thus, only 935 pregnant women were included in the current analysis.

### 2.2. Measurements

#### 2.2.1. Sociodemographic, Anthropometric, and Health-Related Variables

The pregnant women self-reported their sociodemographic variables, including maternal age, gestational age, education, employment status, and whether they had children. The participants also self-reported their last measured body height (cm) and weight (kg) during pregnancy. In addition, the health-related variables, including current smoking status and history of having chronic diseases, were self-reported by each pregnant woman.

#### 2.2.2. Sleep Quality and Duration Evaluation

The Arabic version of the Pittsburgh Sleep Quality Index (PSQI) was used to evaluate sleep quality and duration [23]. This questionnaire has been validated among pregnant women [24]. The PSQI comprises nine items, which are integrated into seven components, including sleep efficiency, sleep disturbance, sleep latency, use of sleep medication, daytime dysfunction, subjective sleep quality, and sleep duration. The score of each component ranges from 0 to 3 points. The total score across all components is aggregated to calculate the global index, ranging from 0 to 21. Using an established scoring method [25], pregnant women were classified as having optimal sleep quality if they scored <5 points on the global index, or having poor sleep quality if they scored ≥5 points. The participants were also classified as those who met sleep duration recommendations if they accumulated 7 to 9 h/day of sleep, or did not meet sleep duration recommendations if they accumulated <7 or >9 h/day of sleep [26]. The sleep duration was estimated using a single self-report item on the PSQI.

#### 2.2.3. Mentally Active and Passive SB Evaluation

The Arabic version of the Sedentary Behavior Questionnaire (SBQ) was used to estimate time spent (h/day) in mentally active and passive SB [27]. This instrument has also been validated among pregnant women [28]. The SBQ queries the time spent (h/day) in nine types of SB on a weekday and, separately, on a weekend day. The SB types include sitting and driving a car, bus, or train, doing paperwork or computer work, sitting and reading a book or magazine, doing artwork or crafts, playing a musical instrument, sitting and talking on the phone, playing computer or video games, watching TV, and sitting and listening to music [29]. These types of SB were then classified according to the previously proposed framework of SB classifications [17], which distinguishes between mentally active and passive SB. Accordingly, watching TV and sitting and listening to music were classified as mentally passive SB because they does not involve higher-order thinking skills. The remaining SB types (sitting and driving a car, bus, or train, doing paperwork or computer work, sitting and reading a book or magazine, doing artwork or crafts, playing a musical instrument, sitting and talking on the phone, playing computer or video games) were classified as mentally active SB, as they demand advanced cognitive processing. Of note, other modern mentally active or passive SB, such as social media use while sitting or playing electronic games on smartphones, were not captured by the SBQ.

Mentally active SB on a weekday was computed by summing the time spent in all types of mentally active SB during a weekday, while mentally active SB on a weekend day was calculated by summing the time spent in all types of mentally passive SB during a weekend day. Then, mentally active SB per day was computed as: mentally active SB per day = ([mentally active SB during a weekday × 5] + [mentally active SB on a weekend day × 2])/7. Lastly, mentally passive SB on weekdays, weekends, and across the week were calculated in the same way but based on mentally passive SB types.

#### 2.2.4. Moderate-to-Vigorous Physical Activity Evaluation

Recent research suggests that moderate-to-vigorous physical activity (MVPA) may influence sleep health and SB in pregnant women [30] and, thus, should be adjusted for in statistical analyses. As such, MVPA was estimated using the Arabic short version of the International Physical Activity Questionnaire (IPAQ) [31]. This instrument has also been validated in pregnant women [32]. The IPAQ queries the number of days and minutes per day pregnant women spent performing moderate physical activity (MPA) or vigorous physical activity (VPA) for at least a 10 min bout during the past week. These estimates were subsequently utilized to determine the average daily time (min/day) spent in MVPA.

### 2.3. Statistical Analyses

The sociodemographic, anthropometric, and health-related variables were reported as mean ± standard deviation or frequency and percentage, as appropriate. Mann–Whitney (for continuous variables) or chi-square (for categorical variables) test compared sociodemographic, anthropometric, and health-related variables between pregnant women of AMA vs. YMA. Binary logistic regression models evaluated whether higher mentally active or passive SB was associated with higher odds of poor sleep quality or not meeting the sleep duration recommendations in pregnant women of AMA and YMA, separately. First, simple logistic regression models were fitted associating one predictor at a time (e.g., mentally active or passive SB) with the binary outcome (i.e., poor sleep quality or not meeting the sleep duration recommendations). Subsequently, adjusted logistic regression models added covariate adjustment for the sociodemographic, anthropometric, and health-related variables, MVPA, and the other SB. Lastly, adjusted logistic regression models assessed the interaction effects of mentally active and passive SB and pregnancy trimesters on sleep quality and duration in pregnant women of AMA and YMA, separately, while adding the same covariate adjustments. JASP software (Version 0.15, JASP) was used to perform all analyses. The significance level was set at *p*-value < 0.05.

## 3. Results

Table 1 displays the sociodemographic, anthropometric, and health-related variables of the included pregnant women. They had an average age of 30.0 ± 5.6 years, with one in every four having an advanced maternal age. Most participants held at least a Bachelor’s degree (58.7%), had at least one child (61.9%), were not currently employed (82.8%), and were generally healthy with no history of chronic disease (91.2%). More than half of the participants (77.2%) had poor sleep quality, but they tended to meet the sleep duration recommendations (54.5%).

Table 1 also shows descriptive comparisons of sociodemographic, anthropometric, and health-related variables between pregnant women of AMA vs. YMA. Those of AMA tended to have higher maternal height (159.5 vs. 158.4 cm; *p* = 0.038) and weight (74.4 vs. 67.4 kg; *p* < 0.001), employment rate (26.2% vs. 22.3%; *p* < 0.001), prevalence of chronic disease (13.3% vs. 7.3%; *p* = 0.005), and children (88.4% vs. 53.3%; *p* < 0.001). Although it was not statistically significant, pregnant women of AMA also tended to have lower adherence to prenatal MVPA recommendations compared to pregnant women of YMA (40.0% vs. 47.0%; *p* = 0.064).

Table 2 shows the associations between mentally active and passive SB and sleep quality in both pregnancy groups. Among participants of AMA, only mentally passive SB was paradoxically associated with sleep quality. Each additional hour of mentally passive SB on weekdays was associated with 58.2% and 112.2% higher odds of poor sleep quality among the overall and second-trimester pregnant women, respectively (*p* < 0.05 for both). On the other hand, each additional hour of mentally passive SB on weekends was related to 37.9% and 46.6% lower odds of poor sleep quality among the overall and third-trimester pregnant women, respectively (*p* < 0.05 for both). Conversely, among those of YMA, neither mentally active nor passive SB was associated with sleep quality (*p* > 0.05 for all). No other significant relationships were found.

Furthermore, Table 3 presents the interaction effects of mentally active and passive SB and pregnancy trimesters on sleep quality in pregnant women of AMA and YMA. Significant interaction effects were found between mentally active SB (across the week and on weekdays) and pregnancy trimester for sleep quality (*p* < 0.001 for all) in pregnant women of YMA only. These interaction effects suggest that the patterns of the association between mentally active SB and sleep quality differ across trimesters, particularly among those in their third trimester compared to those in their first trimester. No other significant interaction effects were detected for either of the pregnancy groups.

Table 4 displays the associations between mentally active and passive SB and the odds of not adhering to the sleep duration recommendations in both pregnancy groups. Paradoxical relationships were observed in participants of AMA. Each additional hour of mentally passive SB across the week or on weekdays was associated with a 27.3% to 65.2% higher odds of not adhering to the sleep duration recommendations (*p* < 0.05 for all). Conversely, each additional hour of mentally active SB across the week or mentally passive SB on weekends was associated with a 13.3% to 43.2% lower odds of not adhering to the sleep duration recommendations (*p* < 0.05 for all). These associations were especially apparent among those in their second or third trimester. Further paradoxical associations were observed among those of YMA. Each additional hour of mentally passive SB across the week or on weekdays was associated with a 10.6% to 29.0% higher odds of not adhering to the sleep duration recommendations (*p* < 0.05 for all). In contrast, each additional hour of mentally active SB across the week or on weekends was associated with a 9.3% to 25.3% lower odds of not adhering to the sleep duration recommendations (*p* < 0.05 for all). These associations were especially apparent among those in their first trimester. No other significant associations were observed.

Moreover, Table 5 shows the interaction effects of mentally active and passive SB and pregnancy trimesters on sleep duration recommendations in pregnant women of AMA and YMA. Significant interaction effects were found between mentally active and passive SB (across the week and on weekdays) and pregnancy trimester for sleep duration recommendations (*p* < 0.001 for all) in pregnant women of YMA only. These interaction effects indicate that the patterns of the association between mentally active and passive SB and sleep quality differ across trimesters, particularly among those in their second or third trimester compared to those in their first trimester. No other significant interaction effects were observed for either of the pregnancy groups.

## 4. Discussion

This investigation is, to our knowledge, the first to examine the associations of mentally active and passive SB with sleep health in pregnant women of AMA and YMA separately, and evaluate the influence of SB patterns. ‘Paradoxical’ associations were detected as discussed in detail below. Collectively, the results suggest that while mentally passive SB on weekdays was associated with poor prenatal sleep health, mentally passive SB on weekends and mentally active SB across the week or on weekends were associated with better sleep health. These relationships were more apparent in pregnant women of AMA for sleep quality and showed a trend with increasing gestational age. However, the cross-sectional design of the study limits the ability to conclude causality or temporality.

### 4.1. Associations of Maternal Mental Activity-Based SB with Sleep Quality and Duration

Research examining the relationships between SB and maternal sleep health beyond measures of total duration or weekly averages remains largely unexplored. The only existing study within this paradigm demonstrated that higher total or leisure SB on weekends was associated with poorer sleep quality but better adherence to sleep duration recommendations in pregnant women (≥18 years old) [16]. Moreover, higher total or leisure SB on weekdays was linked to poorer sleep duration adherence; greater commuting SB, especially on weekends, was related to better sleep duration adherence [16]. To expand this evidence, the present study uniquely investigated the novel associations of mentally active and passive SB (across the week, on weekdays, or weekends) with sleep health in pregnant women of AMA and YMA, separately. Higher mentally passive SB on weekdays was unfavorably associated, and on weekends was favorably associated with sleep quality only in pregnant women of AMA. Furthermore, higher mentally passive SB across the week or on weekdays was unfavorably associated, while higher mentally passive SB on weekends and mentally active SB across the week or on weekends were favorably associated with a higher adherence to sleep duration recommendations among both pregnancy groups. These associations tended to be more apparent in mid-to-late stages of pregnancy. Together, these mixed cross-sectional findings suggest the existence of varying associations of types (mentally active vs. passive), domains (leisure, occupational, commuting), and patterns (weekdays vs. weekends) of SB with sleep health in overall pregnant women and, separately, among those of AMA or YMA. Yet further longitudinal research and randomized controlled trials are warranted to confirm the causal relationships.

Interestingly, the present study detected that higher mentally passive SB on weekends was associated with slightly better maternal sleep outcomes, which may seem ‘paradoxical’ compared with the associations reported for mentally passive SB on weekdays. Given the scarcity of prior research on the relationships between mental activity-based SB and maternal sleep health, these apparently ‘paradoxical’ results should be interpreted cautiously. Several potential hypotheses have been proposed to explain these observed disparities. For instance, a weekend recovery effect, also known as weekend catch-up sleep, could have allowed pregnant women to compensate for their impaired sleep quality during weekdays by extending their sleep duration and taking more naps on weekends [33]. In addition, reverse causation offers another possible explanation for the reported discrepancies; poorer weekday sleep, which has been documented among pregnant women [34], may increase mentally passive SB on weekdays, and vice versa [35]. Unmeasured confounders, including psychosocial stress that tends to be higher on weekdays [36], may also contribute to both higher mentally passive SB and poorer sleep outcomes [37,38], potentially influencing the observed associations. Still, these hypothesized explanations have not been confirmed, especially in pregnant women, and require further examination in future research.

With respect to mentally active SB, accumulating research indicates that high-cognitively demanding SB, especially that includes higher-order thinking tasks, may improve neural connectivity within the frontoparietal control (central executive) network in adults with and without sleep issues [39,40], leading to enhanced sleep continuity, stability, and organization [41,42]. This assumption is moderately supported by the current findings in pregnant women, which revealed that higher mentally active SB on weekends was associated with better adherence to sleep duration recommendations, but not sleep quality. Two recent randomized controlled trials demonstrated that greater mentally active SB (i.e., positive or neutral story reading or reading a book) before sleeping decreased sleep latency, increased sleep duration, and promoted sleep quality in young and older non-pregnant adults [18,19]. The slight discrepancy observed between studies may reflect variations in the types of mentally active SB (e.g., playing computer or video games, reading a book or a story) assessed or differences in the study populations (e.g., young vs. older adults, pregnant vs. non-pregnant individuals). Although the findings remain partially inconsistent, these observations suggest that some types of mentally active SB may influence sleep health in pregnant and non-pregnant adults. Future research should consider the types of mentally active SB measured and the study populations to better understand the associations of mental activity-based SB and maternal sleep health.

### 4.2. Associations of Maternal Mental Activity-Based with Sleep Quality and Duration by Maternal Age

Of particular interest, although the associations of mental activity-based SB with sleep duration were broadly comparable in both pregnancy groups, the paradoxical associations of mentally passive SB with sleep quality were found only among pregnant women of AMA, but not YMA. Pregnant women of AMA regularly undergo a reduction in deep sleep [43], potentially due to declining melatonin levels [9] and elevated inflammation [8], thereby impairing sleep quality. In parallel, pregnant women of AMA are likely to be burdened with greater work/family obligations [44], which can cause irregular sleep patterns and schedules [45]. When combined with the impacts of mentally passive SB, these factors may increase the likelihood of poor sleep quality in this population. On the other hand, pregnant women of YMA may be more capable of counteracting these negative influences by engaging in higher-intensity PA, as they have demonstrated higher PA pre- and during pregnancy compared to AMA [46]. This hypothesis is also supported by the present study, which shows that pregnant women of AMA tended to present with significantly higher rates of chronic disease (possibly greater inflammation), had more children and employment rates (likely more work-family conflicts), and were less likely to meet PA recommendations during pregnancy (borderline significant). This evidence suggests that the contributing factors for sleep quality are distinct in pregnant women of AMA vs. YMA, and mentally passive SB plays a role specifically in those of AMA. However, confirmation of these hypotheses requires further investigations with stronger methodological designs.

### 4.3. Strengths and Limitations

The present study has several strengths that are worth highlighting. First, it involved a large sample size, ensuring adequate statistical power to detect genuine associations, thereby enhancing the precision of the estimates and increasing the generalizability of the results [47]. The latter point was further supported by the comprehensive recruitment strategy implemented, which enrolled pregnant women from all stages of pregnancy with various sociodemographic statuses and from several major Saudi cities. In addition, the SB instrument (i.e., SBQ) utilized estimated time spent in several types of SB, allowing the capture of a wide range of mentally active and passive SB. Measuring important covariates, such as MVPA and sociodemographic variables, and statistically adjusting for them is another strength, as it enables the adjustment for the influence of these covariates.

Nonetheless, the results should be interpreted with caution due to several limitations of the current study, as follows. The cross-sectional design of the study prevents inference of causality or directionality between maternal SB and sleep outcomes. For example, pregnant women may have accumulated more mentally passive SB, such as TV viewing, due to difficulty falling asleep, and the reverse may also be true. The study was unable to determine such directionality. In addition, because there are currently no existing devices capable of measuring mentally active and passive SB, all data were self-reported and, thus, subject to recall and social desirability biases. Moreover, the temporal relationship between weekday/weekend SB and sleep was not directly measured or verified by objective monitoring. Therefore, developing objective tools that can capture mentally active and passive SB, as well as incorporating objective measures of sleep outcomes such as actigraphy or accelerometers, is necessary to obtain more accurate results and to facilitate the assessment of temporal relationships. The SBQ also did not capture modern SBs (e.g., social media use, smartphone screen time), which may have limited its ecological validity [29]. Thus, future studies should also consider using tools that capture a broader range of contemporary SB.

Furthermore, the study population, which was only women residing in Saudi Arabia, mostly unemployed, and healthy, limits the generalizability of the results to diverse or higher-risk populations. Hence, the validity of the associations between mentally active and passive SB with maternal sleep health should be further investigated in more diverse, multicultural populations, including working pregnant women and those at higher risk for comorbidities. Although several covariates were adjusted for, other potential residual confounders (e.g., psychosocial stress, caffeine consumption, parity, work schedule, or exposure to blue light at night) may influence observed associations between maternal SB and sleep outcomes. For instance, higher psychosocial stress may be correlated with higher mentally passive SB, such as TV viewing, and poorer sleep outcomes [37,38]. This could inflate or obscure the true associations reported in the study. Future research should account for such potential residual confounders. Lastly, even though the study has a relatively large sample size (*n* = 935), which improves the statistical power, the multiple testing performed across different models and subgroups may increase the likelihood of type I error. Therefore, this limitation should be considered when interpreting the current findings.

### 4.4. Clinical Implications

This cross-sectional study observed paradoxical associations of mentally active and passive SB with prenatal sleep health. Mentally active SB was favorably associated, while mentally passive SB, specifically on weekdays, was unfavorably associated with sleep health, especially among pregnant women of AMA. Although these findings suggest potential links between prenatal mental activity-based SB and sleep outcomes, especially in pregnancies with a higher age-related risk, the cross-sectional nature of the study does not allow for causal conclusions. As a result, the translation of these results into clinical practice should be considered cautiously. Longitudinal studies and randomized clinical trials that implement more inclusive mental activity-based SB assessment tools are needed to determine the causal relationships between mentally active and passive SB with sleep outcomes in pregnant women of AMA and YMA.

## 5. Conclusions

Expanding the existing literature, the present study revealed that while mentally passive SB on weekdays was associated with poorer prenatal sleep health, mentally passive SB on weekends and mentally active SB across the week or on weekends were associated with better sleep health. Noticeably, the relationships found were particularly evident in pregnant women of AMA for sleep quality and showed a trend with increasing gestational age. Nevertheless, the cross-sectional design of the study limits the ability to draw causal conclusions. These findings encourage further longitudinal studies and randomized trials examining the causal influence of mentally active and passive SB on sleep health during pregnancy, separately in those of AMA and YMA.

## Figures and Tables

**Table 1:** Sociodemographic, anthropometric, and health-related variables in pregnant women.

Variable	Overall Sample (*n* = 935)	Pregnant Women of AMA (*n* = 225)	Pregnant Women of YMA (*n* = 710)	*p*-Value for Between-Group Comparisons
	Mean ± SD, *n* (%)			
Maternal Age (years old)	30.0 ± 5.6	**37.8 ± 2.6**	**27.5 ± 3.8**	**<0.001 #**
Maternal Height (cm)	158.7 ± 6.4	**159.5 ± 6.7**	**158.4 ± 6.3**	**0.038 #**
Maternal Weight During Pregnancy (kg)	69.1 ± 14.7	**74.4 ± 14.4**	**67.4 ± 14.3**	**<0.001 #**
Gestational Age				0.589 *
First Trimester	225 (24.1)	59 (26.2)	166 (23.4)	
Second Trimester	317 (33.9)	71 (31.6)	246 (34.6)	
Third Trimester	393 (42.0)	95 (42.2)	298 (42.0)	
Education				<0.001 *
Diploma or Less	386 (41.3)	**106 (47.1)**	**280 (39.4)**	
Undergraduate	520 (55.6)	**104 (46.2)**	**416 (58.6)**	
Postgraduate	29 (3.1)	**15 (6.7)**	**14 (2.0)**	
Currently Employed				<0.001 *
No	774 (82.8)	**166 (73.8)**	**552 (77.7)**	
Yes	162 (17.2)	**59 (26.2)**	**158 (22.3)**	
Currently Smoking				0.978 *
No	914 (97.8)	220 (97.8)	694 (97.7)	
Yes	21 (2.2)	5 (2.2)	16 (2.3)	
History of Chronic Disease				0.005 *
No	853 (91.2)	**195 (86.7)**	**685 (92.7)**	
Yes	82 (8.8)	**30 (13.3)**	**52 (7.3)**	
Have Children				<0.001 *
No	356 (38.1)	**26 (11.6)**	**330 (46.5)**	
Yes	579 (61.9)	**199 (88.4)**	**380 (53.5)**	
Mentally Active SB (h/day)	3.5 ± 2.6	3.6 ± 2.5	3.5 ± 2.7	0.293 #
Mentally Passive SB (h/day)	3.3 ± 2.2	3.1 ± 2.2	3.3 ± 2.2	0.187 #
Sleep Quality	7.7 ± 3.7	7.7 ± 3.7	7.7 ± 3.7	0.992 # 0.892 *
Optimal Sleep Quality	213 (22.8)	52 (23.1)	161 (22.7)	
Poor Sleep Quality	722 (77.2)	173 (76.9)	549 (77.3)	
Sleep Duration (h/day)	7.2 ± 2.1	7.1 ± 2.0	7.3 ± 2.1	0.236 # 0.615 *
Meet Sleep Duration Recommendations	510 (54.5)	126 (56.0)	384 (54.1)	
Do Not Meet Sleep Duration Recommendations	425 (45.5)	99 (44.0)	326 (45.9)	
MVPA (min/day)	32.1 ± 48.2	32.4 ± 58.4	32.0 ± 44.6	0.232 # 0.064 *
Meet MVPA Recommendations	424 (45.3)	90 (40.0)	334 (47.0)	
Do Not Meet MVPA Recommendations	511 (54.7)	135 (60.0)	376 (53.0)	

AMA: advanced maternal age; cm: centimeter; h: hours; kg: kilogram; min: minutes; MVPA: moderate-to-vigorous physical activity; *n*: number; SB: sedentary behavior; SD: standard deviation; YMA: younger maternal age. # indicates Mann–Whitney test. * indicates chi-square test. Bold indicates significant association (*p* < 0.05).

**Table 2:** Odds ratios of poor sleep quality per h/day of mentally active and passive sedentary behavior (SB) in pregnant women of advanced (AMA) and young (YMA) maternal age.

Variables	Mentally Active SB (H/Day)	Mentally Passive SB (H/Day)	Mentally Active SB (H/Weekday)	Mentally Passive SB (H/Weekday)	Mentally Active SB (H/Weekend Day)	Mentally Passive SB (H/Weekend Day)
	AMA (≥35 Years)					
All Trimesters (*n* = 225)						
OR (95% confidence interval)	1.112 (0.968, 1.277)	1.090 (0.926, 1.282)	1.129 (0.957, 1.331)	**1.582** **(1.197, 2.091)**	1.053 (0.885, 1.253)	**0.621** **(0.461, 0.837)**
First Trimester (*n* = 59)						
OR (95% confidence interval)	1.188 (0.863, 1.635)	1.115 (0.818, 1.522)	1.692 (0.916, 3.125)	1.952 (0.881, 4.326)	0.712 (0.427, 1.187)	0.550 (0.244, 1.238)
Second Trimester (*n* = 71)						
OR (95% confidence interval)	0.993 (0.729, 1.352)	1.335 (0.857, 2.081)	1.113 (0.775, 1.599)	**2.122** **(1.056, 4.265)**	1.018 (0.657, 1.576)	0.534 (0.254, 1.121)
Third Trimester (*n* = 95)						
OR (95% confidence interval)	1.140 (0.876, 1.485)	0.948 (0.723, 1.243)	1.128 (0.840, 1.515)	1.436 (0.959, 2.150)	1.254 (0.919, 1.711)	**0.534** **(0.317, 0.901)**
YMA (<35 years old)						
All Trimesters (*n* = 710)						
OR (95% confidence interval)	0.987 (0.924, 1.053)	0.988 (0.912, 1.071)	0.978 (0.902, 1.059)	0.927 (0.839, 1.024)	0.999 (0.914, 1.091)	1.090 (0.975, 1.219)
First Trimester (*n* = 166)						
OR (95% confidence interval)	0.872 (0.741, 1.025)	0.937 (0.776, 1.131)	0.864 (0.713, 1.046)	0.877 (0.681, 1.129)	0.992 (0.815, 1.207)	1.097 (0.821, 1.466)
Second Trimester (*n* = 246)						
OR (95% confidence interval)	0.976 (0.878, 1.086)	0.999 (0.864, 1.156)	0.957 (0.829, 1.104)	0.929 (0.786, 1.098)	1.010 (0.867, 1.177)	1.105 (0.913, 1.337)
Third Trimester (*n* = 298)						
OR (95% confidence interval)	1.069 (0.961, 1.190)	0.994 (0.882, 1.122)	1.057 (0.929, 1.202)	0.894 (0.770, 1.039)	0.994 (0.856, 1.154)	1.157 (0.969, 1.382)

AMA: advanced maternal age; h: hours; *n*: number; OR: odd ratio; SB: sedentary behavior; YMA: younger maternal age. All models were adjusted for age, smoking status, moderate-to-vigorous physical activity, having children, education, occupation, and chronic disease status, and simultaneously adjusted for the other mental activity-based SB. Bold indicates significant association (*p* < 0.05).

**Table 3:** Interaction effects of mentally active and passive sedentary behavior (SB) (h/day) and pregnancy trimesters on sleep quality in pregnant women of advanced (AMA) and young (YMA) maternal age.

Variables	Mentally Active SB (H/Day)	Mentally Passive SB (H/Day)	Mentally Active SB (H/Weekday)	Mentally Passive SB (H/Weekday)	Mentally Active SB (H/Weekend Day)	Mentally Passive SB (H/Weekend Day)
	AMA (≥35 Years) (*n* = 225)					
SB × Pregnancy Trimester Interaction [Second (*n* = 71) vs. First (*n* = 59)] OR (95% confidence interval)	0.873 (0.602, 1.265)	1.015 (0.656, 1.572)	0.814 (0.568, 1.166)	0.939 (0.616, 1.432)	1.052 (0.738, 1.499)	0.971 (0.631, 1.493)
SB × Pregnancy Trimester Interaction [Third (*n* = 95) vs. First (*n* = 59)] OR (95% confidence interval)	0.948 (0.661, 1.358)	0.795 (0.539, 1.172)	0.852 (0.597, 1.216)	0.688 (0.466, 1.015)	1.197 (0.875, 1.637)	0.864 (0.570, 1.312)
YMA (<35 years old) (*n* = 710)						
SB × Pregnancy Trimester Interaction [Second (*n* = 246) vs. First (*n* = 166)] OR (95% confidence interval)	1.121 (0.934, 1.345)	1.084 (0.869, 1.353)	1.106 (0.935, 1.303)	1.072 (0.875, 1.313)	1.081 (0.910, 1.285)	1.101 (0.883, 1.371)
SB × Pregnancy Trimester Interaction [Third (*n* = 298) vs. First (*n* = 166)] OR (95% confidence interval)	**1.212** **(1.009, 1.456)**	1.104 (0.897, 1.358)	**1.197** **(1.014, 1.413)**	1.075 (0.889, 1.299)	1.131 (0.946, 1.352)	1.171 (0.947, 1.447)

AMA: advanced maternal age; h: hours; *n*: number; OR: odd ratio; SB: sedentary behavior; YMA: younger maternal age. All models were adjusted for age, smoking status, moderate-to-vigorous physical activity, having children, education, occupation, and chronic disease status, and simultaneously adjusted for the other mental activity-based SB. Bold indicates significant interaction effects between SB and pregnancy trimester on sleep quality (*p* < 0.05).

**Table 4:** Odds ratios of not adhering to sleep duration recommendations per h/day of mentally active and passive sedentary behavior (SB) in pregnant women of advanced (AMA) and young (YMA) maternal age.

Variables	Mentally Active SB (H/Day)	Mentally Passive SB (H/Day)	Mentally Active SB (H/Weekday)	Mentally Passive SB (H/Weekday)	Mentally Active SB (H/Weekend Day)	Mentally Passive SB (H/Weekend Day)
	AMA (≥35 Years)					
All Trimesters (*n* = 225)						
OR (95% confidence interval)	**0.867** **(0.769, 0.977)**	1.136 (0.994, 1.297)	0.973 (0.847, 1.118)	**1.273** **(1.052, 1.540)**	0.896 (0.770, 1.042)	0.837 (0.675, 1.038)
First Trimester (*n* = 59)						
OR (95% confidence interval)	0.817 (0.618, 1.079)	0.936 (0.698, 1.254)	0.903 (0.616, 1.322)	0.831 (0.491, 1.409)	0.905 (0.629, 1.304)	1.144 (0.664, 1.970)
Second Trimester (*n* = 71)						
OR (95% confidence interval)	0.923 (0.743, 1.147)	1.092 (0.833, 1.430)	1.134 (0.856, 1.503)	**1.652** **(1.048, 2.602)**	0.860 (0.611, 1.212)	**0.568** **(0.345, 0.935)**
Third Trimester (*n* = 95)						
OR (95% confidence interval)	**0.786** **(0.618, 0.999)**	**1.309** **(1.025, 1.673)**	0.970 (0.750, 1.254)	**1.529** **(1.047, 2.234)**	0.844 (0.650, 1.095)	0.741 (0.474, 1.158)
YMA (<35 years old)						
All Trimesters (*n* = 710)						
OR (95% confidence interval)	**0.907** **(0.855, 0.962)**	**1.106** **(1.032, 1.185)**	1.012 (0.943, 1.087)	1.057 (0.967, 1.155)	**0.869** **(0.802, 0.942)**	1.046 (0.948, 1.153)
First Trimester (*n* = 166)						
OR (95% confidence interval)	**0.747** **(0.637, 0.876)**	**1.290** **(1.087, 1.530)**	0.901 (0.755, 1.075)	**1.289** **(1.021, 1.627)**	**0.819** **(0.678, 0.989)**	0.983 (0.767, 1.260)
Second Trimester (*n* = 246)						
OR (95% confidence interval)	0.949 (0.863, 1.044)	1.010 (0.890, 1.145)	1.016 (0.892, 1.157	0.937 (0.805, 1.091)	0.911 (0.795, 1.044)	1.109 (0.936, 1.314)
Third Trimester (*n* = 298)						
OR (95% confidence interval)	0.938 (0.857, 1.027)	1.089 (0.982, 1.208)	1.074 (0.966, 1.194)	1.077 (0.941, 1.234)	**0.826** **(0.725, 0.942)**	0.982 (0.843, 1.143)

AMA: advanced maternal age; h: hours; *n*: number; OR: odd ratio; SB: sedentary behavior; YMA: younger maternal age. All models were adjusted for age, smoking status, moderate-to-vigorous physical activity, having children, education, occupation, and chronic disease status, and simultaneously adjusted for the other mental activity-based SB. Bold indicates significant association (*p* < 0.05).

**Table 5:** Interaction effects of mentally active and passive sedentary behavior (SB) (h/day) and pregnancy trimesters on sleep duration recommendations in pregnant women of advanced (AMA) and young (YMA) maternal age.

Variables	Mentally Active SB (H/Day)	Mentally Passive SB (H/Day)	Mentally Active SB (H/Weekday)	Mentally Passive SB (H/Weekday)	Mentally Active SB (H/Weekend Day)	Mentally Passive SB (H/Weekend Day)
	AMA (≥35 Years) (*n* = 225)					
SB × Pregnancy Trimester Interaction [Second (*n* = 71) vs. First (*n* = 59)] OR (95% confidence interval)	1.130 (0.829, 1.542)	1.398 (0.990, 1.972)	1.108 (0.827, 1.483)	1.379 (0.993, 1.916)	1.059 (0.785, 1.430)	1.203 (0.847, 1.709)
SB × Pregnancy Trimester Interaction [Third (*n* = 95) vs. First (*n* = 59)] OR (95% confidence interval)	0.999 (0.725, 1.377)	1.401 (0.988, 1.986)	1.014 (0.748, 1.375)	1.348 (0.967, 1.880)	0.941 (0.702, 1.262)	1.223 (0.852, 1.755)
YMA (<35 years old) (*n* = 710)						
SB × Pregnancy Trimester Interaction [Second (*n* = 246) vs. First (*n* = 166)] OR (95% confidence interval)	**1.203** **(1.015, 1.427)**	**0.820** **(0.674, 0.997)**	**1.182** **(1.011, 1.383)**	**0.804** **(0.670, 0.965)**	1.176 (0.995, 1.391)	0.945 (0.784, 1.140)
SB × Pregnancy Trimester Interaction [Third (*n* = 298) vs. First (*n* = 166)] OR (95% confidence interval)	**1.210** **(1.022, 1.433)**	0.874 (0.728, 1.049)	**1.179** **(1.013, 1.371)**	0.868 (0.732, 1.030)	1.113 (0.937, 1.322)	0.937 (0.784, 1.120)

AMA: advanced maternal age; h: hours; *n*: number; OR: odd ratio; SB: sedentary behavior; YMA: younger maternal age. All models were adjusted for age, smoking status, moderate-to-vigorous physical activity, having children, education, occupation, and chronic disease status, and simultaneously adjusted for the other mental activity-based SB. Bold indicates significant interaction effects between SB and pregnancy trimester on sleep duration recommendations (*p* < 0.05).

## Data Availability

The data are available upon reasonable request from the corresponding author.

## Abbreviations

The following abbreviations are used in this manuscript:

AMA	Advanced Maternal Age
IPAQ	International Physical Activity Questionnaire
MPA	Moderate Physical Activity
MVPA	Moderate-to-Vigorous Physical Activity
*n*	Number
OR	Odd Ratio
PA	Physical Activity
PSQI	Pittsburgh Sleep Quality Index
SB	Sedentary Behavior
SBQ	Sedentary Behavior Questionnaire
STROBE	The Strengthening the Reporting of Observational Studies in Epidemiology
VPA	Vigorous Physical Activity
YMA	Young Maternal Age
